# Successful implementation of interprofessional education: A pedagogical design perspective

**DOI:** 10.12688/mep.20331.2

**Published:** 2025-08-27

**Authors:** Alex Lepage-Farrell, Anne Marie Pinard, Amélie Richard

**Affiliations:** 1 Department of Pediatrics, London Children's Hospital, London, Ontario, Canada; 2 University of Western Ontario, London, Ontario, Canada; 3 Department of Anesthesiology and Intensive Care, University Laval, Québec, Quebec, Canada; 4 Department of Preschool and Elementary Teaching, University of Sherbrooke, Sherbrooke, Quebec, Canada

**Keywords:** Interprofessional collaboration, interprofessional education, residency, postgraduate training, pedagogical design

## Abstract

Interprofessional collaboration (IPC) is crucial within healthcare teams that must provide safe and quality care to their patients. Competent professionals in this area offer better care and contribute to a medical culture where IPC and teamwork are valued. To become competent, they must be adequately trained. The literature describes that, unfortunately, collaboration training is uneven across professions. Interprofessional education (IPE) could fill this educational gap but remains challenging to implement. This article aims to present ten clear and concise considerations to implementing IPE initiatives successfully, following a well-described pedagogical designing process. After reading, the clinician-educator will be informed of the newest evidence in IPE as well as the common pitfalls to avoid. From the starting point of a recent synthesis article on IPE, several additional syntheses, analyses, and recommendations articles were consulted and synthesized. From that, the findings are organized according to the “ADDIE” model, a flexible methodology used in pedagogical design through iterative cycles in context. The phases of “ADDIE” are analysis, design, development, implementation, and evaluation. According to these phases, the considerations will be presented to allow the reader to apply them "step by step" in their educational planning process. Ten considerations are presented, from the needs analysis, stakeholders and Faculty involvement, composition of the design team, selection of students and types of learning activity, the role of reflexivity, training of facilitators, supervision, and the continuous improvement process. Taken together, these will contribute to highlighting the essential nature of training in collaboration in modern professionalizing programs.

## Introduction

Interprofessional collaboration (IPC) is essential amongst healthcare teams, who must provide safe, high-quality patient care. IPC is described as the joint action of healthcare professionals with their patients, their families, and the community, that aims to provide the best care ^1^. The evidence supporting IPC is growing: healthcare professionals who work in collaboration can give better care and contribute to a culture in healthcare where collaboration is encouraged, and teamwork is satisfying ^2^. Beyond benefiting healthcare professionals, interprofessional collaboration is also associated with better patient outcomes ^3^. Therefore, being competent in collaboration and understanding its fundamental principles is essential for any healthcare worker.

## Background

Interprofessional education (IPE) happens when two or more learners or groups of learners from different professions learn with, about, and from each other ^4, 5^. Through IPE, learners can better understand the roles of other professionals and develop the necessary competencies to be good collaborators ^6^, as well as a favorable attitude towards collaboration ^7, 8^. The current literature describes that different professionals are unevenly trained for IPC, even though, in practice, they all collaborate daily and recognize its importance ^6^.

Concepts of IPC and IPE are tightly linked at micro-, meso-, and macrosystemic levels ^9– 12^, as described in numerous systematic reviews. The growing literature shows that IPE could be a valuable way to teach collaboration, especially in emergency settings ^13^. Even if collaboration competency is now an objective for accreditation organisms, inserting IPE in current healthcare faculty curricula faces many obstacles ^14^, at all levels of implementation ^12, 15^. Competing interests, heavy workloads, and limited resources are all factors dissuading educators who are otherwise willing to plan new and innovative teaching opportunities.

**Figure 1.**
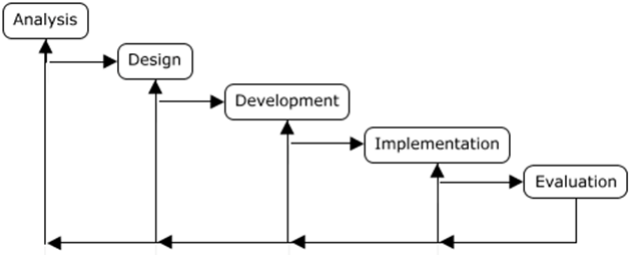
Representation of the ADDIE model, adapted from Molenda (2003), including the iterative steps of analysis (A), design (D), development (D), implementation (I), and evaluation (E).

Beyond these obstacles to implementing IPE, the fundamental step of planning such teaching activities tends to be challenging. While IPE curriculum may find grounds in many different education theories ^16^, few articles explain how to specifically apply pedagogical design to IPE. Pedagogical design is multifaceted. As a theoretical concept in education sciences, pedagogical design aims at constructing knowledge about instruction (or education), from its strategies to its application, efficiently and optimally ^17^. As a process, pedagogical design can also describe planning the steps and the conception of an educational system, based on current educational theories ^18^. Here is where the notion of educational planning as a method emerges. One frequently used method is the “ADDIE” model ( Figure 1), a classic approach ^19^. The name “ADDIE” is used to describe a method that allows a systematic approach to pedagogical planning through a five-step “iterative” model: analysis, design (or conception), development, implementation, and evaluation ^20^. It is a relatively easy-to-use system for novice educators looking to develop any educational activity. It is also a way to ensure the presence of an essential element of pedagogical design: coherence. Coherence, or constructive alignment, happens when the conception of the activity (from the objectives, the activity, and the evaluation) gravitates around the end goals of the training program ^21^. The analysis phase of the “ADDIE” model happens when the needs analysis is performed, to identify the gap between the actual educational situation and the desired one. The design (or conception) phase happens when you determine the activity’s content, its sequence, strategies, and teaching methods, always minding the learning objectives previously identified. The development phase is the more practical one of the models where the different teaching tools and materials are produced. The implementation phase represents the moment when the activity is introduced to the learners. This phase ultimately leads to the evaluation phase when the assessment of the learners and the teaching activity (through evaluation) takes place ^22, 23^.

## Goal and objectives

Recommendations exist to guide medical educators who want to implement IPE in their respective departments or clinical workplaces. Somehow, few recommendations are presented based on a realistic pedagogical process that would happen when developing an evidence-based teaching activity. This article aims to summarize clear and concise considerations in IPE planning that highlight what works and how to avoid the common pitfalls of IPE, following the well-documented pedagogical design process “ADDIE” to facilitate its implementation.

## Methods

The current literature was reviewed in two main steps. The starting point was a recent review article aiming to document the different IPE initiatives already described in the literature ^24^. From this review, after consulting the articles it references, we then searched additional research articles and systematic reviews. From this pragmatic literature review, articles that were included provided significant supplemental recommendations following the best pedagogical planning practices. The different findings were analyzed, then summarized into main overarching themes. The goal of this article is to provide guidance on IPE implementation following a structured pedagogical planning process. Therefore, the themes were ordered following a logical order, presented according to the steps of the “ADDIE” model.

## Tips

### Step 1 of ADDIE: Analysis

***Do your homework: observe and analyze the educational context.*** Identify the organization’s needs, resources, and goals (including the competing ones), as the essential details influencing IPE implementation reside within its targeted educational context ^11, 25^. Once all the above factors have been considered, rank the program's needs based on the highest priority ^26^. A thorough analysis also includes the meso- and macrosystemic influencers (at an organizational and political level), which go beyond the microsystemic level ^9, 27^. We think a good understanding of these factors will empower educators to adapt their projects to the needs expressed and allow early identification of catalysts and incoming obstacles to change. It will also help identify if a particular competency framework could be suited for the program, which could then be used as the foundation of the initiatives, starting with its learning objectives. Elaborating learning objectives is a crucial step into maintaining educational coherence throughout the pedagogical design process. The Canadian Interprofessional Health Collaborative framework is an example of a framework currently informing the Canadian programs in IPE ^1^.

***Be strategic: get institutional commitment and external resources.*** Top-down support from faculty leadership is necessary to plan the teaching activities with current politics in mind and establish common goals and objectives for the learning initiative ^28, 29^. Engagement of the institutional stakeholders is also essential as it could lead to a collaborative culture that transcends beyond just teaching activities. It should be included in an institutional strategic plan and a clear IPE mission should be established ^29^. A strategic approach should also include external support planning; government bodies, foundations, funding organisms, or private resources should be looked at for financial support. One significant and common limiting factor in IPE implementation is material resources – these should be planned for early ^11^. We encourage developing a small and low-cost initiative for programs that are less familiar with IPE.

***Be well surrounded: set a team that makes the dream work.*** To develop an IPE initiative, you must assemble a team representing the different groups impacted by your activities. Such groups include clinical teams, managers, patients, community members, faculty representatives, professional representant, and even students ^30^. Recruitment of volunteer and enthusiastic professionals is essential in early IPE champion recognition ^31^. No resources to our knowledge specify how many should be on that team but literature does show that the initiative’s leadership should be shared amongst professionals from different backgrounds ^25, 32^. That way, the entire process, from the development to the implementation and evaluation, models professional collaboration. That team will maintain good communication with the institution and stakeholders, from planning to implementation to results ^26^. This ensures an efficient coalition and a clear vision for the project.

***Connect your faculty to reality: create a collaborative community.*** The faculty leaders should be kept informed of the clinical reality through constant and mutual communication with the clinical learning environment. This connection maintains a relationship where reciprocal development is emphasized ^14^. When adequately done, the clinical field benefits from up-to-date knowledge and best practices, and, in return, faculty leaders can get feedback and keep in touch with the reality of clinical activities. It also is an excellent opportunity for faculty development and networking ^2, 25, 32^. Finally, good communication between the clinical, faculty, and research fields is essential to align science with policy ^33^. That way, new learning initiatives can stem from their clinical context. The evaluation process can serve both quality improvement and contribute to the global knowledge on IPE and IPC.

### Step 2 of ADDIE: Design

***Adapt to your learners: plan the right content for the right students.*** Choosing the right content, or activities, is crucial in pedagogical design. Your choices should be based on the learners' needs and their existing competencies, the abilities of the teachers, and the IPE objectives identified in the analysis phase. Different taxonomies are suggested in the literature, notably the UBC model, which associates the students’ ability levels to suggestions of appropriate activities ^4, 5^. Some other models and examples have been more recently published and provide a good foundation for activity planning ^29, 34, 35^. It is essential to consider the learners’ training level in pedagogical design because their professional identity is flourishing and a careless choice could lead to adverse outcomes ^36^. For example, postgraduate students better understand who they are as professionals and their role in the healthcare system. The activities directed towards them should then foster reflective discussion and a better understanding of their contribution, as well as others, to the healthcare system ^36^. They could also be positively challenged in that dual identity (theirs as a professional that’s also part of a team) ^37, 38^. It is also essential to choose the right students to group. A common mistake in pedagogical planning is to make an inappropriate pairing, which could lead to groups being too far apart in their knowledge or needs, leading to negative experiences ^14, 39^. We believe a tailored approach could maximize students' buy-in.

### Step 3 of ADDIE: Development

***Teach wisely: prepare the appropriate teaching strategies.*** Many types of activities are suggested for IPE ^24^, and it could easily get confusing for an educator new to this field. The current consensus is that there is no true superiority to any modality ^40^ if the choice is coherent with the learners' needs and goals. In general, small groups offer more flexibility and allow more engagement from the learners ^41^. The activities should let the students experiment while being assisted in constructing meaningful learning and provide models of IPC ^2, 30^. Also, practical activities in a real healthcare environment should be prioritized, and experiential learning should happen through activities that foster good practice in collaborative work ^25^.

***Foster awareness: the crucial role of reflectivity.*** It is crucial to incorporate a reflective component in any activities where learners perform clinical and interprofessional tasks ^31^, in a transferable and transversal way ^30^. Learners should be supported in every step of the reflectivity process, through thorough event analysis covering the circumstances, why they happened, and each actor’s perspective ^42^. The reasoning should be made explicit, as skilled facilitators will be able to challenge their students' beliefs and have them ultimately build together a shared understanding of the patient’s needs ^43^, even discussing how the patient’s may benefit from IPC. Different methods are suggested to include reflectivity to IPE. Some literature suggests small case analysis, feedback, and facilitation ^44^. Reflection-on-action is also a good option, where facilitated discussions are held right after a clinical situation has been encountered ^42^. Carefully integrated discussions are a great way for supervisors to position themselves as collaborative role models while allowing the learners to think collaboratively about their actions as individuals part of a team (and those of others), the challenges faced and possible solutions ^2^.

***Teach the teachers: support the facilitators.*** Supervisor’s training is recognized as one of the determining factors in the IPE experience for learners and comes from training and participation in IPE ^45^. As described recently in a recent report on sustainability of teaching in IPE, facilitators need to feel recognized, confident, and inspired ^46^. Unfortunately, teachers could find it difficult to supervise and facilitate IPE without exposure to it in their training days ^29^, or if local resources in IPE are lacking. That is why continuous support through a community of supervisors or even interprofessional co-teaching is essential. It allows supervisors to ask questions, share successes and challenges and think of solutions together ^31, 47^. Then, supervisors’ training should gravitate around developing the essential competencies needed to support interprofessional teams: interprofessional dialogue, conflict management, evaluation of collaborative competencies, and reflective practice ^31, 47^.

### Steps 4 and 5 of ADDIE: Implementation and Evaluation

***Don’t leave feedback behind: choose the right tools for learners’ assessment.*** Assessment modalities should be chosen coherently with the programs’ objectives, the teaching methods, and the intensity of the clinical tasks ^2, 48^. In an authentic setting, interprofessional supervision should also be prioritized and planned early on 49. Many assessment tools have been identified and validated for IPE, although many were only studied with doctors or nurses. No matter what is ultimately chosen, aligning the tools with what is being assessed is crucial ^21^. Uniform tools, applicable to various professional groups, are also ideal ^49, 50^ and are described in the literature ^51, 52^. Such tools could allow multiple supervisors from different professional backgrounds to conduct the assessment process, regardless of the learners' profession, just as long as the results are evaluated against the program's goals ^53^. Supervision from many professionals is an interesting option that could allow more flexibility and alleviate the assessment burden in some contexts where supervisors are limited. In some studies, such interprofessional supervision led to safer care while reinforcing the collaborative and non-hierarchical climate that IPE seeks ^54^.

***Better initiatives through evaluation: quality improvement.*** The IPE evaluation process should be mapped in the early stages of planning, even though it mostly happens towards the end of an iteration in practice. It is an essential methodological step in planning and decisions should be made coherently with the primary learning goals ^30^. Big or small initiatives may even include a “pilot”, allowing for quick feedback and adjustment. IPE evaluation can be done through various methods; however, the Kirkpatrick or Kirkpatrick-Barr propositions are some of the most often utilized ^55, 56^. One other well-documented method is the design-based research (DBR) methodology. DBR is a systematic research methodology integrating design and flexibility to improve educational initiatives by conducting iterative analysis in an authentic context ^57^. Any kind of continuous evaluation should allow learners' evaluation but should also provide perspective on the initiative and its impact on the different involved groups as well as on the supervisors, then on the patients and the organization, to adjust as needed ^11, 30, 33^. This should then lead to revision of any deficient component. Evaluation reports can also demonstrate the activity’s “plus value” to key stakeholders who should be kept informed ^58^. To improve the quality of education, it is essential to continue documenting the effects of IPE on patient care, through an evaluation process that should be well documented ^33^.

## Conclusion

Collaboration amongst healthcare workers is crucial in providing good patient care; interprofessional learning activities represent a promising way to foster collaboration competencies. Implementation of interprofessional education is complex, and many challenges were identified in the current literature, including resources management, community and learner buy-in, as well as content and teaching activity selection, to only name a few. Pedagogical methodology can be applied to interprofessional education and ensure coherence through all the steps leading to its implementation. Once the learning context is carefully analyzed and the appropriate teaching method is chosen, the critical factors in efficient and successful IPE implementation include early and strategic engagement of stakeholders and faculties and judicious implementation, including reflexivity and sound assessment methods. Finally, a continuous quality improvement process should allow knowledge dissemination. That, put together, could surely put forward the essential nature of IPE in modern training curricula.

## Data Availability

There is no data associated with this article.
