# Supplementary material for: Successful implementation of interprofessional education: A pedagogical design perspective
Source: MedEdPublish (2016). 2025 Aug 27;14:55. Originally published 2024 Jul 12. [Version 2] doi: 10.12688/mep.20331.2 (PMC11480705; doi:10.12688/mep.20331.2)
Supplement: Peer review history

# Reviewer report: Reviewer response for version 2

**DOI:** 10.21956/mep.22774.r43270

**Published:** 2025-09-02

**Authors:** Lyn Gum

**Affiliations:** 1 University of South Australia, Adelaide, South Australia, Australia

No further comments to make, thank you.

If evidence from practice is presented, are all the underlying source data available to ensure full reproducibility?

Yes

Is the topic of the practical tips discussed accurately in the context of the current literature

Yes

Are all factual statements correct and adequately supported by citations?

Yes

Are the conclusions drawn balanced and justified on the basis of the presented arguments?

Yes

Are arguments sufficiently supported by evidence from the published literature and/or the authors’ practice?

Yes

Reviewer Expertise:

PhD - IPE and collaborative practice

I confirm that I have read this submission and believe that I have an appropriate level of expertise to confirm that it is of an acceptable scientific standard.

# Reviewer report: Reviewer response for version 1

**DOI:** 10.21956/mep.21761.r38984

**Published:** 2024-10-15

**Authors:** Thomas Chacko

**Affiliations:** 1 Believers Church Medical College, Kerala, India

The authors have used a scholarly approach by doing adequate preparation by using current literature and appropriate frameworks in the field of Instructional Design / Pedagogical planning process  and Inter Professional Education and collaborative practice to come up with the ten literature supported considerations to be addressed within the steps of the ADDIE framework.

 However, in order to make the considerations complete and in sequence , some of the considerations listed under the **Development** and **Implementation** components of the A-D-D-IE  framework can be revisited and some more elements for consideration under **Implementation**  like piloting the designed IPE course to identify the "loose threads" would make the manuscript even better in guiding new IPE implementation teams embarking on the journey of planning - implementation and evaluation for quality improvement.

If evidence from practice is presented, are all the underlying source data available to ensure full reproducibility?

Yes

Is the topic of the practical tips discussed accurately in the context of the current literature

Yes

Are all factual statements correct and adequately supported by citations?

Yes

Are the conclusions drawn balanced and justified on the basis of the presented arguments?

Yes

Are arguments sufficiently supported by evidence from the published literature and/or the authors’ practice?

Yes

Reviewer Expertise:

Have been guiding Fellows undergoing Faculty Leadership Fellowship which requires design and evaluation of  Educational Innovations

I confirm that I have read this submission and believe that I have an appropriate level of expertise to confirm that it is of an acceptable scientific standard.

# Response

**Published:** 2025-08-20

**Authors:** Alex Lepage-Farrell

**Affiliations:** Cardiovascular Intensive Care, Cincinnati Children's Hospital, Cincinnati, Ohio, USA

Hi, Thank you for these comments. Please find the revised version of the article, in which you will find adjustments with regards to describing the "Evaluation" phase of the ADDIE model. The recommendation to mention "piloting" was very useful and included in the newly submitted version.  Thank you very much for the consideration while reviewing our work.

# Reviewer report: Reviewer response for version 1

**DOI:** 10.21956/mep.21761.r38734

**Published:** 2024-10-07

**Authors:** Moni Fricke, Laura MacDonald

**Affiliations:** 1 Rady Faculty of Health Sciences, University of Manitoba, Winnipeg, Manitoba, Canada; 2 Dental Hygiene, University of Manitoba Faculty of Health Sciences, Winnipeg, Manitoba, Canada

The submission is timely and important in fostering sustainable IPE. 

 The authors offer recommendations using a pedagogical framework (ADDIE) summarized from  some of the published literature. Readers will find valuable tips for the construction and sustainability of IPE opportunities. 

**We have a few comments:**

**Methods**

 The tips provided by the authors (their finding) draw upon some of the literature, particularly from published papers already providing tips (e.g., reference 15, 35, 38, 29). In the methodology section, the authors need to clarify the process employed in arriving at the selected papers. For e.g., search engines, key words, inclusion and exclusion criteria. Importantly, others should be able to reproduce the search.

 The use of ADDIE, the pedagogical design/framework, is interesting and quite helpful. We suggest that the authors clearly state that they used ADDIE to process/organize the tips garnered from the literature; it is unclear as written. We see this as a unique process. The authors might consider integrating the literature reviews (scoping, systematic, meta-analysis) included in their paper into the introduction/background or the discussion (e.g., reference 7, 8, 10, 11, 12, etc.). 

**Literature**

- One of the goals of the paper was to identify common pitfalls. The authors need to clearly state these pitfalls and do so within the ADDIE framework, given it is the organizational process they are employing. This will help the reader.
- In step 1, the authors mention the importance of using a competency framework. This step would be strengthened by linking it to theory underpinning IPE (e.g., Hean S., et al., 2018).
- In step 2, the authors mention double identity, please see dual identity (Khalili, 2022). The authors may want to include mention of longitudinal curricula (Azzam et al., 2023). 

The reference need attention as some are incomplete, e.g., #23.

 The conclusion is concise. 

 Importantly, grammar and sentence structure needs attention as it is distracting to content coherency.

 The authors remind us to use frameworks.

If evidence from practice is presented, are all the underlying source data available to ensure full reproducibility?

Partly

Is the topic of the practical tips discussed accurately in the context of the current literature

Partly

Are all factual statements correct and adequately supported by citations?

Partly

Are the conclusions drawn balanced and justified on the basis of the presented arguments?

Yes

Are arguments sufficiently supported by evidence from the published literature and/or the authors’ practice?

Partly

Reviewer Expertise:

Interprofessional education and collaboration (pedagogy, curriculum design)

We confirm that we have read this submission and believe that we have an appropriate level of expertise to confirm that it is of an acceptable scientific standard, however we have significant reservations, as outlined above.

## References

[1] : The contribution of theory to the design, delivery, and evaluation of interprofessional curricula: BEME Guide No. 49. *Med Teach* .2018;40(6) : 10.1080/0142159X.2018.1432851 542-558 10.1080/0142159X.2018.1432851 29457926

[2] : From uniprofessionality to interprofessionality: dual vs dueling identities in healthcare. *J Interprof Care* .2022;36(3) : 10.1080/13561820.2021.1928029 473-478 10.1080/13561820.2021.1928029 34139953

[3] : A Case Study of Organizational and Curricular Attributes for Interprofessional Education: A Model for Sustainable Curriculum Delivery. *Journal of Research in Interprofessional Practice and Education* .2023;13(1) : 10.22230/jripe.2023v13n1a351 10.22230/jripe.2023v13n1a351

# Response

**Published:** 2025-08-20

**Authors:** Alex Lepage-Farrell

**Affiliations:** Cardiovascular Intensive Care, Cincinnati Children's Hospital, Cincinnati, Ohio, USA

Hi, Thank you very much for these insightful comments. This author has reviewed the manuscript accordingly and wanted to answer a few questions/comments:

* Methods:  The methodology section has been revised, adding clarity to the review process and changes were made to the first sections to place more emphasis on the pertinence of using the ADDIE model. 

* Literature: Some elements were reorganized through the different steps to highlight common mistakes while planning for IPE, although the group expressly wanted to mostly formulate positive recommendation. All three references were reviewed and included in the revised version of the manuscript.

 Thank you very much for these suggestions, as they were very interesting reads and valuable addition to our work. 

* General comments: The references were adjusted and updated, and grammar was reviewed. 

 Again, thank you very much for the consideration while reviewing our work. Please see the revised version of the article.

# Reviewer report: Reviewer response for version 1

**DOI:** 10.21956/mep.21761.r37950

**Published:** 2024-08-09

**Authors:** Lyn Gum

**Affiliations:** 1 University of South Australia, Adelaide, South Australia, Australia

An excellent, well researched resource.

 I have some comments for the authors to consider.

**Abstract/Background:**

 The word ‘unfortunately’ in the sentence about current literature reads as is if its unfortunately regarding the literature itself rather than the problem of not having equal training. The sentence needs revising, can authors be clear about what is the actual issue, e.g. that it is inopportune when it comes to IPC?

**Introduction/Background:**

 The authors describe IPC quite well. However, there is a difference between the terms or concepts of ‘interprofessional collaboration’ and ‘collaborative practice’. IPC is quite broad, whereas collaborative practice emphasises the practice aspect and what competency in collaboration would entail. For example, mentioning the need to be aware of the principles that make teamwork and/or collaborative practice amongst the multiple professionals in a team, effective, such as mutual trust, shared decision-making, communication skills and role clarity.

**ADDIE steps -**

***Foster awareness: the crucial role of reflectivity***

- Authors mention how through perspective there is shared understanding of the patient’s needs. However, could be slightly expanded here for readers to also consider how this shared understanding means recognising how the patient themselves will benefit from the IPC.

***Teach the teachers: support the facilitators.***

- “Unfortunately, teachers could find it difficult to supervise and facilitate IPE without exposure to it in their training years ^28^, or if they feel isolated and helpless.”
- This sentence reads quite negatively. Rather authors could focus or emphasise what would be required of a facilitator. There are certain attributes required - which could be mentioned to demonstrate how it’s important to ensure that there should be suitable facilitators recruited for the role.

***Don’t leave feedback behind: choose the right tools for learners’ assessment.***

- “Assessment modalities should be chosen coherently with the programs’ objectives, the teaching methods, and the intensity of the clinical tasks”
- This is an important aspect but seems limited. There are various purposes for assessing which could be outlined for the reader.
- Not sure if the authors agree but wondering - would it be helpful to give the readers some examples of common or popular tools and in what type of contexts/type of activities they could be used?

**General comment:**

- Authors talk about pedagogical methodology in relation to interprofessional education. My understanding of this concept would be a blend of practical and theoretical insights. I am not sure of the reason, but there is nothing mentioned about theoretical perspective to guide the design and planning of IPE. This might be seen as a limitation of the 10 considerations?
- Thank you for the opportunity to review this article. I have enjoyed reading this evidence-based resource that can further inform IPE design.

If evidence from practice is presented, are all the underlying source data available to ensure full reproducibility?

Yes

Is the topic of the practical tips discussed accurately in the context of the current literature

Yes

Are all factual statements correct and adequately supported by citations?

Yes

Are the conclusions drawn balanced and justified on the basis of the presented arguments?

Yes

Are arguments sufficiently supported by evidence from the published literature and/or the authors’ practice?

Yes

Reviewer Expertise:

PhD - IPE and collaborative practice

I confirm that I have read this submission and believe that I have an appropriate level of expertise to confirm that it is of an acceptable scientific standard.

# Response

**Published:** 2025-08-20

**Authors:** Alex Lepage-Farrell

**Affiliations:** Cardiovascular Intensive Care, Cincinnati Children's Hospital, Cincinnati, Ohio, USA

Hi,  

 Thank you very much for this detailed response.  

 Some minor modifications have been made to the manuscript in response and this author wanted to answer some questions:

 * ***Abstract/Background: *** Modifications have been made to ensure clarity.

 * ***Introduction/Background:*** This is indeed a very insightful comment, and a small precision was made in the revised version. 

 * ***Foster awareness: the crucial role of reflectivity:*** Thank you for this useful suggestion, adding depth to the recommendation. 

 * ***Teach the teacher: support the facilitators:*** Slight modification has been made to convey the appropriate idea. 

 * ***Don't leave the feedback behind: choose the right tools for the learners' assessment: *** Some valuable tools for collaborative competency assessment are presented in the in-text references. Due to the limitations of word count, not all tools could be described and the authors felt like providing references would be enough. The same decision was made in prior recommendations, notably with regards to content and teaching activity planning. 

 * ***General comment:*** The spirit of the article was to keep it very "practical", sort of a basic reference for educators who would like to get started in IPE, and present recommendations in a step-wise approach that fits a well known pedagogical process. This is why the group has stayed away from diving into more of the theoretical perspectives of pedagogical design for IPE, as we though it may defeat the purpose.   Again, thank you for the consideration while reading this article. Please enjoy the newly submitted revision.
